# Plain Talk, but No Offense

**Published:** 1877-09-20

**Authors:** 


PLAIN TALK, BUT NO OFFENSE.—To those who have paid their subscriptions to THE RECORD we are thankful; but certain parties who have received the journal on short indulgence have failed to respond, after frequent reminders, even so far as to encourage us with a promise of *any intention to pay at all.* Of course no *gentleman* will refuse to pay after taking the journal from the office for several months. Our continuance of THE RECORD to these parties, under the circumstances, is therefore proof of our confidence in the integrity and honor of medical men. Can it be that a single one will disappoint us?

